# Feline cutaneous histoplasmosis in a non-endemic region: diagnostic challenges and molecular confirmation of *Histoplasma capsulatum* in Latvia

**DOI:** 10.1186/s13028-026-00883-4

**Published:** 2026-09-07

**Authors:** Inese Berzina, Madara Nikolajenko, Ilze Matise-VanHoutana, Viktorija Ulanova, Agnija Kivrāne, Renāte Ranka

**Affiliations:** 1 Preclinical Institute, Faculty of Veterinary Medicine, Latvia University of Life Sciences and Technology, Kr. Helmana iela 8, Jelgava, LV-3004 Latvia; 2 Clinical Institute, Faculty of Veterinary Medicine, Latvia University of Life Sciences and Technology, Kr. Helmana iela 8, Jelgava, LV-3004 Latvia; 3 Faculty of Pharmacy, Riga Stradiņš University, Konsula Street 21, Riga, LV-1007 Latvia; 4 Laboratory of Molecular Microbiology, Latvian Biomedical Research and Study Centre, Rātsupītes Street 1, k1, Riga, LV-1067 Latvia

**Keywords:** Autochthonous infection, Cytology, Granulomatous dermatitis, *Histoplasma capsulatum*, Molecular diagnostics, Non-endemic region

## Abstract

**Background:**

Feline histoplasmosis can have variable clinical presentations, it is rarely diagnosed in Europe and has not been diagnosed in Northern Europe and Baltics. We encountered a cluster of autochthonous cases of feline histoplasmosis in Latvia—a non-endemic region. In this paper we describe the diagnostic pathways, challenges and outcomes in three cats. We also report the first molecular confirmation of *Histoplasma capsulatum* in the Baltic region. Three domestic shorthaired cats presented with ulcerative or erosive skin lesions and were initially evaluated by cytology. Diagnostic follow-up included histopathology, immunohistochemistry (IHC), serology and in one case a polymerase chain reaction (PCR), with DNA sequencing and phylogenetic analysis. We outlined several practical challenges of performing fungal diagnostics in Europe, these can be valuable to our colleagues.

**Results:**

All three cases showed organisms consistent with *Histoplasma* species on cytological examination. Case 1: a feline immunodeficiency virus positive shelter cat with multiple ulcerative cutaneous lesions. Histopathology and IHC confirmed fungal infection suggestive of *Histoplasma* species. The cat was lost to follow-up. Case 2: A 3-year-old male cat with a single erosive facial lesion. The cat fully recovered with antifungal treatment. Case 3: A 6-year-old neutered male with multiple erosive skin lesions. The cat was humanely euthanized. Diagnosis was supported by cytology while results of histopathology and IHC were suggestive of *Histoplasma* species. Serology (urine and serum) were positive for *H. capsulatum* antigen and the diagnosis was confirmed by PCR. Sequencing and phylogenetic analysis of the isolate provided the first molecular confirmation of feline histoplasmosis in the Baltic region.

**Conclusions:**

We report the occurrence of clustered autochthonous feline histoplasmosis in Latvia and expand the recognized geographic range of molecularly confirmed disease in Europe. Our cases show the advantages of cytology as an initial diagnostic tool and highlight the utility of serology and molecular methods in regions with limited access to well-established diagnostic methods for non-endemic fungal diseases. Our findings suggest that histoplasmosis may be underdiagnosed in non-endemic areas and reinforce the need for heightened clinical awareness among veterinarians across Europe.

## Background

*Histoplasma capsulatum* is a dimorphic, soil-dwelling fungus that belongs to the phylum *Ascomycota*, class: *Eurotiomycetes* and genus: *histoplasma* [1]. Classically histoplasmosis is considered endemic in the USA, Latin America, parts of Africa, India and Southeast Asia, but recently suspected to be more widespread and potentially endemic in Central Europe, Turkey and Japan [1–4]. Histoplasma are present as moulds in the environment but morph into yeast form upon entering the host [1, 2]. Feline histoplasmosis cases have been reported in Central Europe: Germany, France, Switzerland, Italy and Netherlands [2, 5–9].

Feline histoplasmosis can have variable clinical presentations - vague systemic signs (lethargy, anorexia, weight loss, fever) or manifest with primarily gastrointestinal or pulmonary involvement or disseminated disease (liver, lymph nodes, eyes, bone marrow may be affected) [5, 10, 11]. Occasionally it may manifest exclusively with cutaneous or subcutaneous lesions, which can complicate clinical suspicion and delay diagnosis [5, 8, 12]. An additional diagnostic challenge is posed by the ability of yeast to survive intracellularly within the macrophages; therefore, clinical signs of histoplasmosis can manifest even years after the exposure to the pathogen or disease can be exacerbated by immunosuppressive factors [4]. Careful patient history and general awareness of various clinical signs of histoplasmosis are essential to ensure timely diagnosis and effective treatment [6, 9].

No cases of feline histoplasmosis have been previously reported from Baltic States or Northern Europe. This report describes three autochthonous cases of feline histoplasmosis in Latvia, one of which was confirmed by molecular identification and phylogenetic analysis of *H. capsulatum*. Diagnostic pathways and limitations—especially in regions with restricted access to serology and immunohistochemistry—are also discussed.

The aim of our study is to raise awareness about feline histoplasmosis in Europe, to disseminate the knowledge about the diagnostic options and potential errors in diagnosing this disease.

## Methods

Three domestic shorthaired cats presented to two different referral veterinary clinics in Latvia between 2019 and 2020 for evaluation of ulcerative or erosive cutaneous lesions. Signalment, clinical history, physical examination findings and treatment approaches were recorded. Additionally, we describe the clinical course, sample collection procedures and outcomes for each case.

### Clinical cases

#### Case 1

adult male cat of unknown age was presented to a referral veterinary clinic in Riga in May 2019 after being transferred from a local animal shelter in Tukums city (approximately 70 km away from the capital Riga). The cat tested positive for feline immunodeficiency virus (FIV) and exhibited multiple ulcerative lesions on the head, back and forelimbs (Fig. 1a). Fine-needle aspiration (FNA) and skin biopsy samples were collected from affected areas. Histoplasmosis was highly suspected cytologically, the cat was treated with oral itraconazole (Itranol^®^, 100 mg capsules, Olainfarm, Latvia). In histopathology diagnosis of fungal infection was made with differential diagnoses of *Sporothrix* and *Histoplasma* species based on the morphology of organisms. After two weeks of antifungal therapy, the shelter reported partial improvement in the lesions on the back and legs, while the head lesions showed no change. The cat remained clinically stable and continued to eat and drink normally and was subsequently lost to follow-up.

**Fig. 1a:**
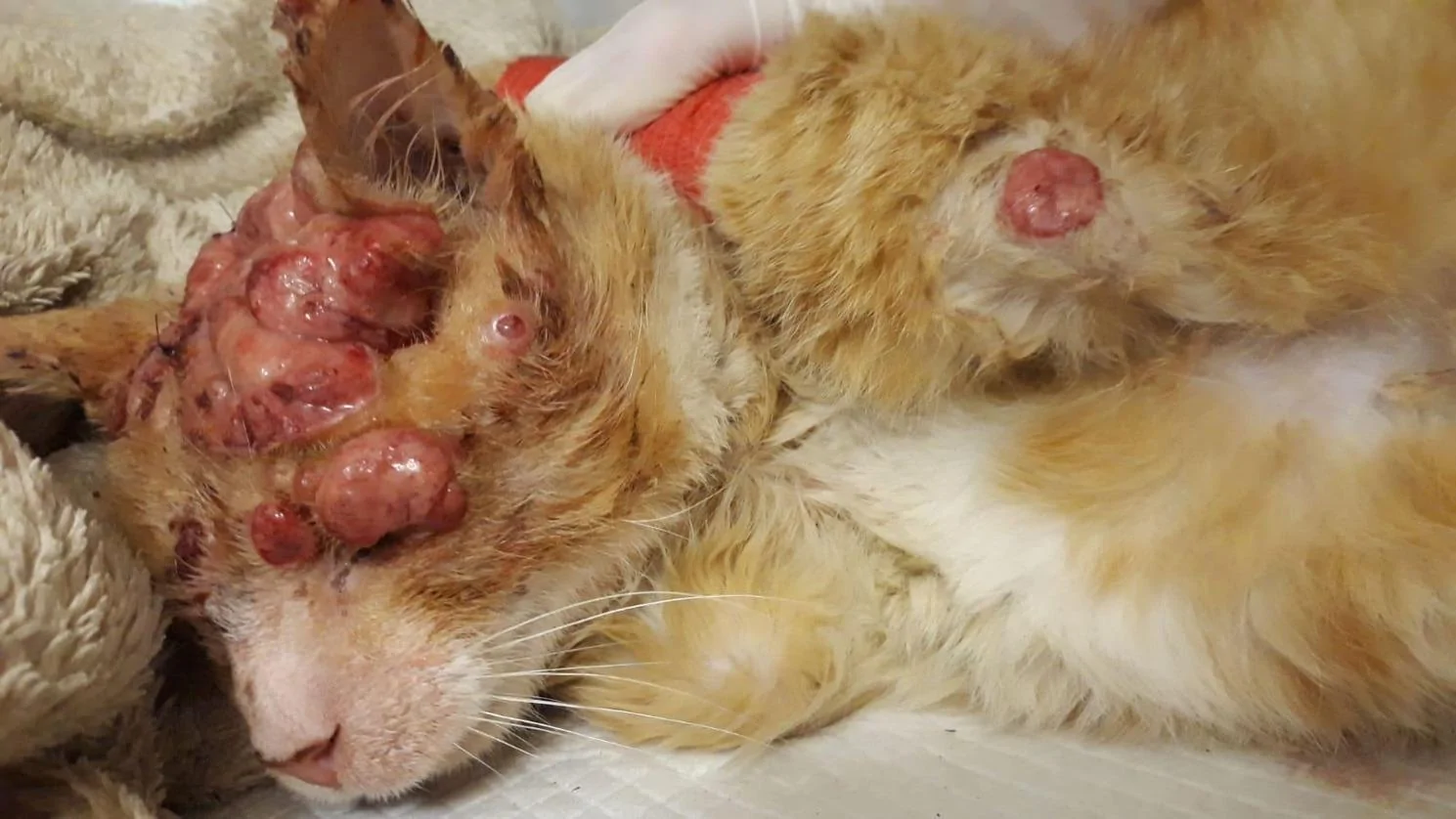
Gross image of cat 1 showing advanced skin changes. Severe, proliferative, nodular lesions of the frontal region. Multiple proliferative, nodular to plaque-like lesions are evident on the forehead, periocular region and the base of the left ear. A similar raised lesion is also visible on the medial aspect of the forelimb. Cytological findings consistent with *Histoplasma capsulatum* infection. The lesions developed rapidly and were the initial clinical sign prompting further diagnostic investigation

#### Case 2

in August 2019, a 3-year-old male domestic shorthaired cat from Tukums was forwarded to the same reference clinic as the Case 1 cat. This patient had a single erosive lesion on the left cheek (Fig. 1b). An FNA was obtained for cytological evaluation that was consistent with histoplasmosis. The cat received oral itraconazole treatment following the same protocol used for Case 1. Clinical follow-up revealed complete resolution of the lesion and the cat made a full recovery (Fig. 1c).

**Fig. 1b:**
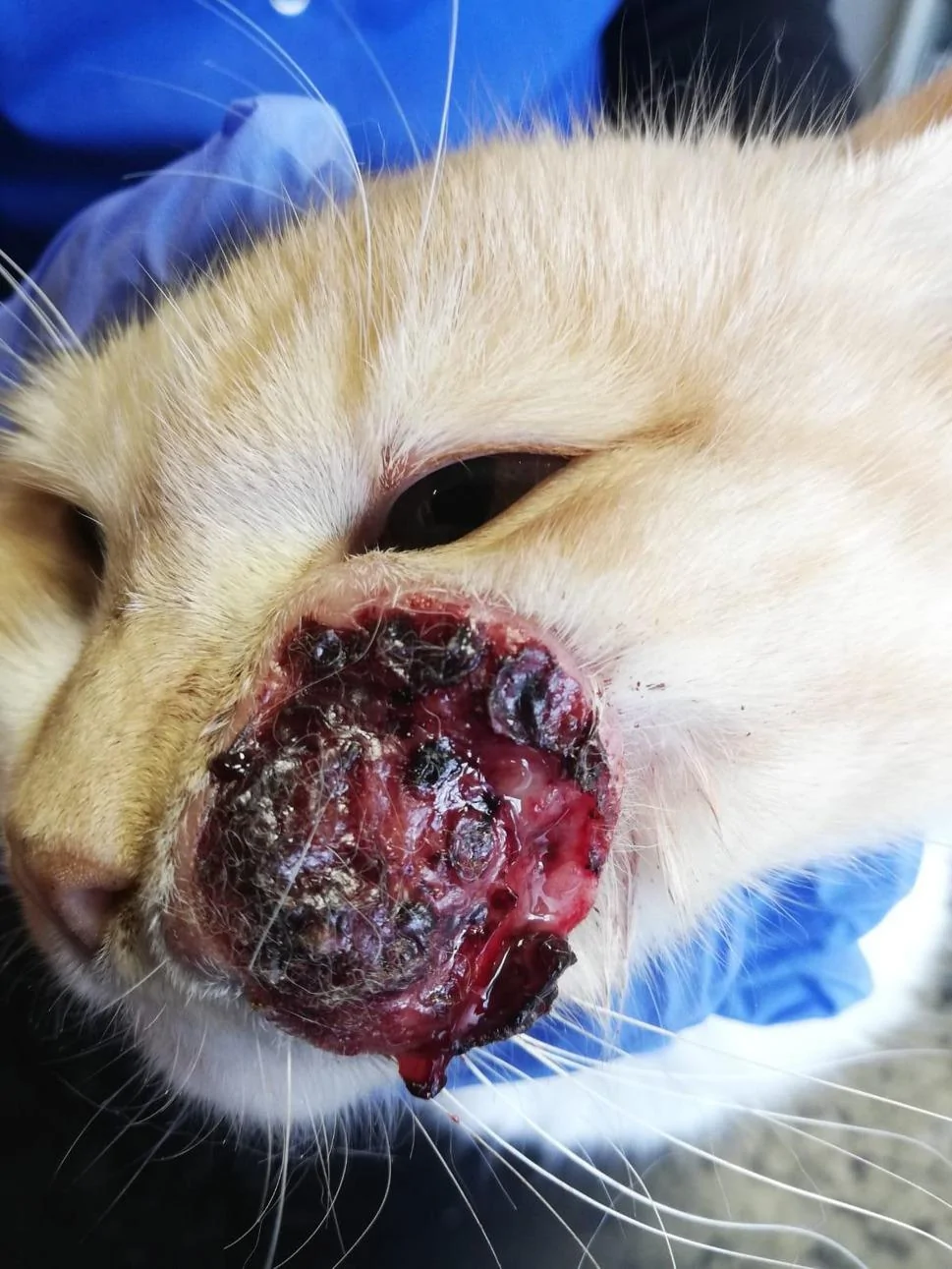
Gross image of skin lesion in cat 2. Large, ulcerated, crusted mass lesion on the left maxilla, before antifungal treatment

**Fig. 1c:**
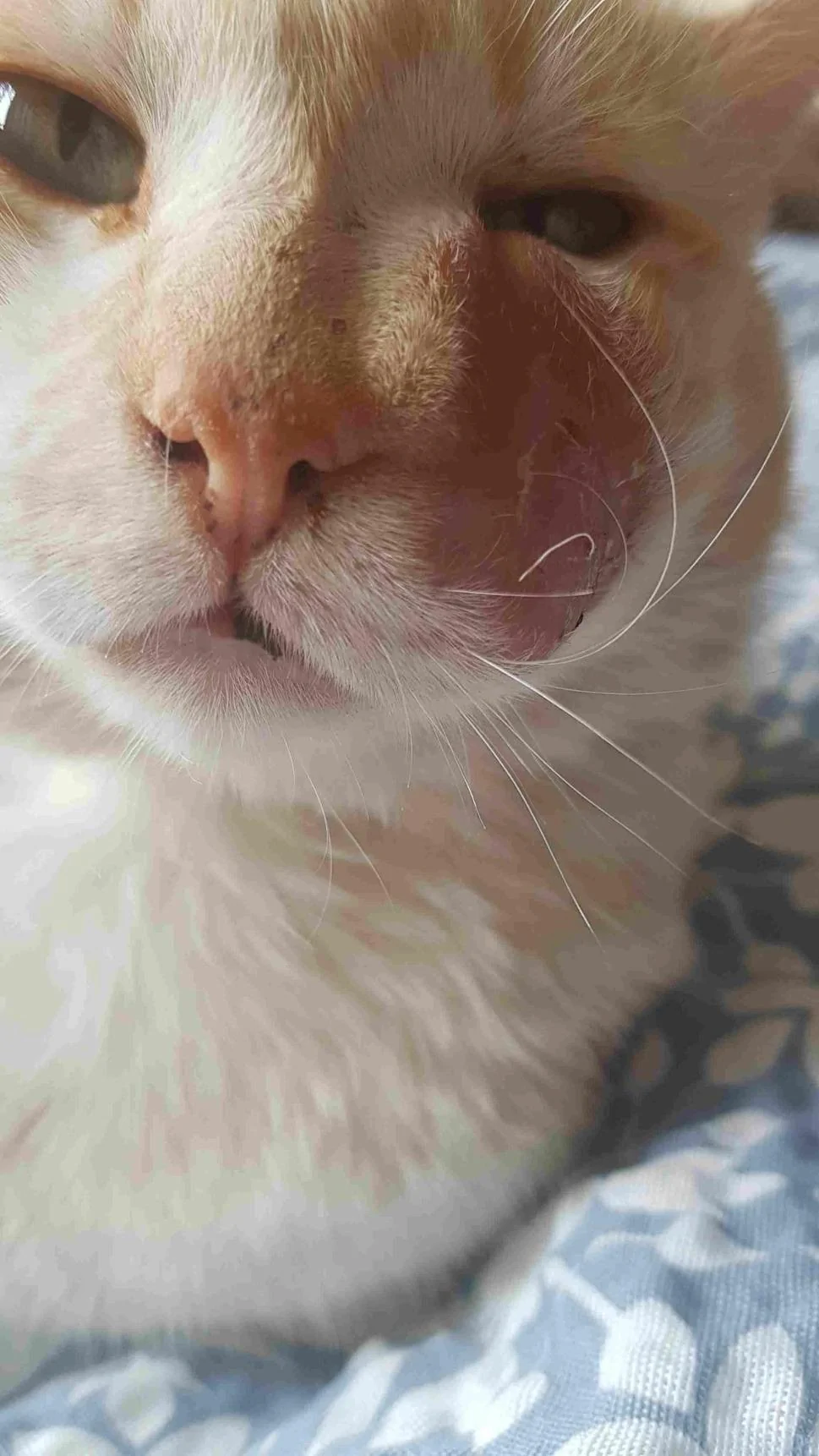
Gross image of lesion resolution in cat 2. Appearance of the left maxillary region 8 weeks after initiation of antifungaltreatment with itraconazole. Marked clinical improvement with lesion resolution and hair regrowth is evident

#### Case 3

in autumn 2020, A 6-year-old castrated male domestic shorthaired cat from Tukums with indoor–outdoor access was presented with multiple erosive and pustular skin lesions on the ears, head and distal limbs (Fig. 1d and 1e). According to the owner, the lesions developed approximately two weeks after a cat fight. Initial management by a local veterinarian included wound cleaning and topical antibiotic treatment with streptomycin ointment, but no clinical improvement was obtained. The cat was referred to a specialty clinic for further evaluation. On presentation, the cat weighed 5 kg and had a body condition score of 3/5. Vital parameters were within normal limits. FNA and impression smears were obtained from the affected skin areas for cytological examination that was consistent with histoplasmosis. The owners declined additional diagnostics, including retroviral testing and bloodwork. After the cytological evaluation, the cat was humanely euthanized. With owners’ informed consent, post-mortem samples; skin biopsies, serum and urine, were collected for further testing at the authors’ expense. In all above described cases best practice standards of veterinary care were followed.

**Fig. 1d:**
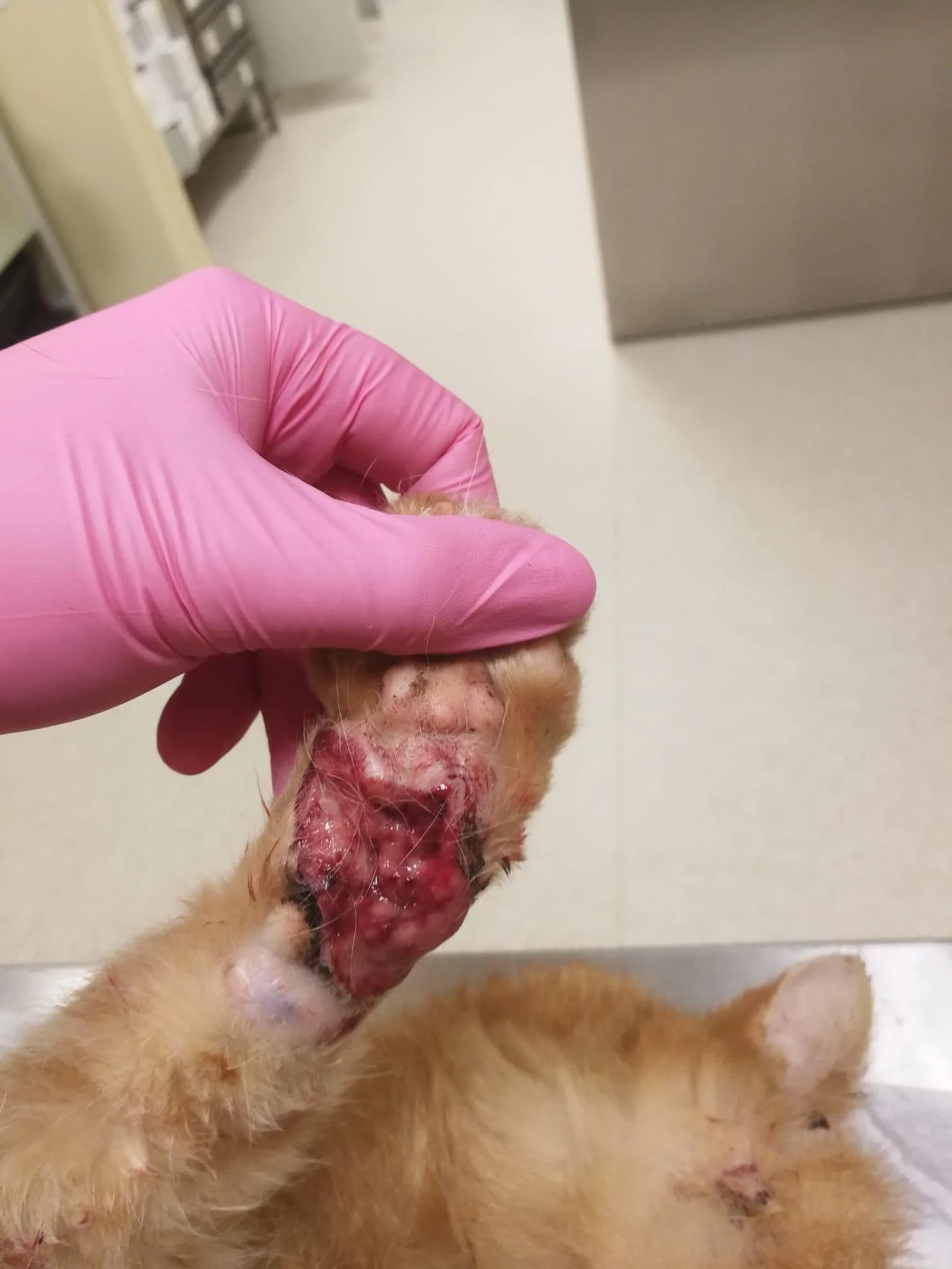
Gross image of cat 3 showing skin lesions on the forelimb. Deep ulcerative lesions with crusting, involving soft tissues of the paw, limbs, head and trunk were similarly affected

**Fig. 1e:**
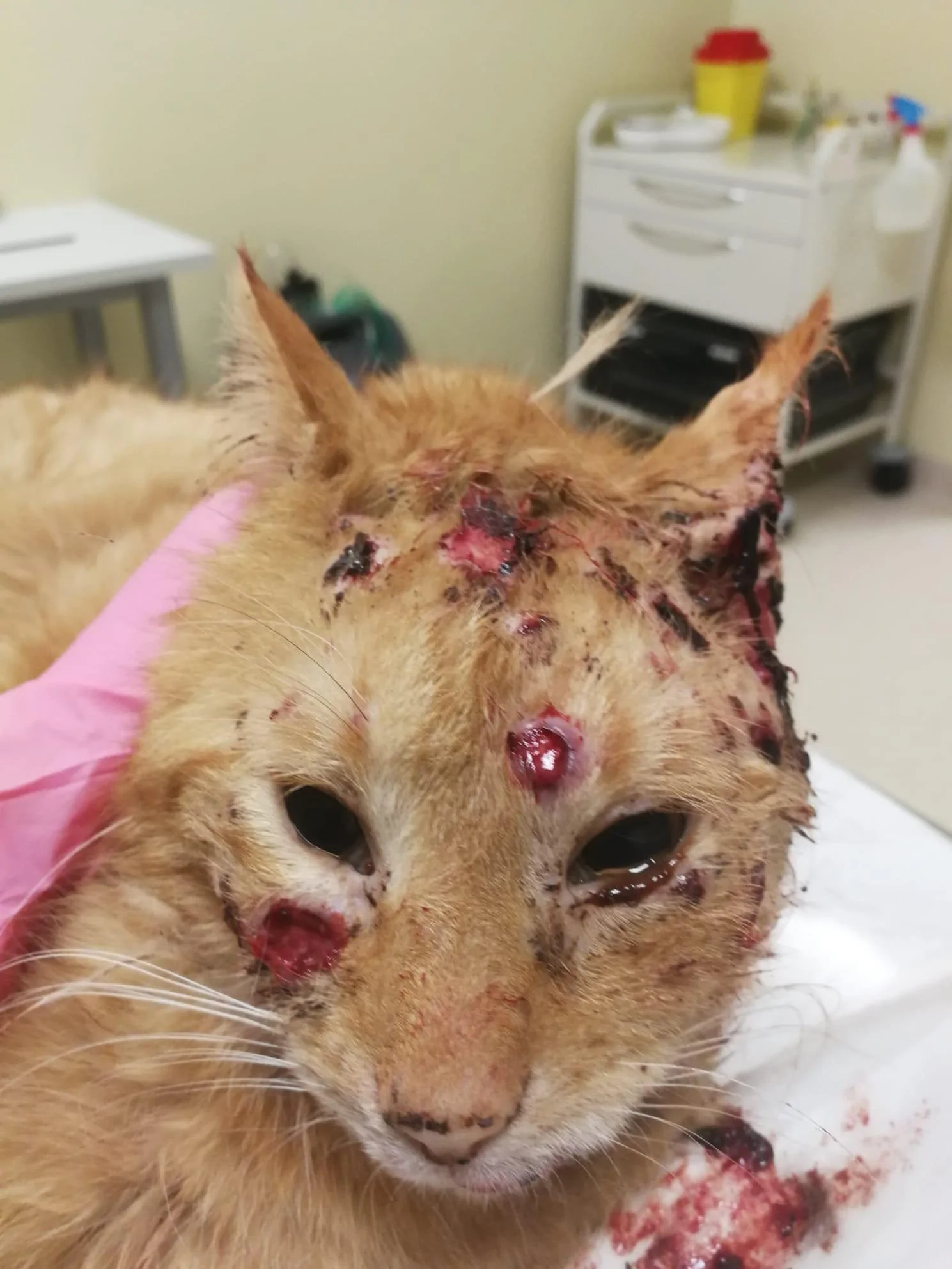
Gross image of cat 3 showing multifocal skin lesions on the head. Crusted, ulcerative and erosive lesions involving theperiocular, nasal and frontal regions, consistent with disseminated fungal infection

### Cytology, histopathology and immunohistochemistry

Cytology – fine needle aspirates and impression smears were stained with a modified Romanowsky stain (Diff-Quik^®^, Jorvet, USA) and examined under light microscope (10×, 20×, 100× objectives). Photomicrographs were obtained using a smartphone camera. Histopathological analysis of formalin-fixed, paraffin-embedded (FFPE) skin sections was performed on hematoxylin and eosin (H&E) stained slides. Histological sections from case 3 were additionally stained with Periodic acid–Schiff (PAS) used for fungal identification. From Cases 1 and 3 FFPE tissue blocks were sent for immunohistochemistry for *Histoplasma* species antigen detection to Michigan State University Veterinary Diagnostic Laboratory.

### DNA extraction and real-time PCR

DNA was extracted from FFPE skin tissue (Case 3) using the QIAamp DNA FFPE Advanced Kit (Qiagen, Germany) according to the manufacturer’s instructions. Two eluates were collected during extraction and labelled H1 and H2. Positive control DNA was extracted from a histology slide of murine tissues inoculated with *H. capsulatum*.

Real-time polymerase chain reaction (PCR) was performed targeting the internal transcribed spacer 1 (ITS1) region of *H. capsulatum* using a TaqMan-based assay. The reaction included TaqMan Fast Advanced Master Mix (Thermo Fisher Scientific, USA), primers HcITS-54 F (5′-ACCCTTGTCTACCGGACCTGTT-3′), HcITS-204R (5′-TTTTGACTGGATTATTATCGCTCTCA-3′) and probe HcITS-155 (5′ 6-FAM-CGGTGAACGATTGGCGTCTGAGC-BHQ-1 3′) [13]. Each 20 µL reaction contained 0.4 µM primers, 0.2 µM probe and 4 µL of extracted DNA. Thermal cycling included UNG pre-treatment at 50 °C for 2 min, denaturation at 95 °C for 20 s, followed by 40 cycles of 95 °C for 3 s and 60 °C for 30 s.

### Nested PCR and sequencing

Nested PCR targeting the 100 kDa protein gene (Hcp100) was performed using a modified method [14]. The primary PCR amplified a 391 bp fragment with primers Hc I and Hc II and the nested PCR amplified a 210 bp fragment using primers Hc III and Hc IV. Reaction conditions included initial denaturation at 95 °C for 10 min, then 35 cycles of 95 °C for 15 s, 58 °C (primary reaction) or 55 °C (nested reaction) for 30 s, 72 °C for 30 s; and a final extension step at 72 °C for 5 min.

The final reaction volume was 25 µL, containing DreamTaq buffer, 1.25 U DreamTaq Hot Start polymerase, 200 µM dNTPs, 0.32 µM primers and 2 µL DNA template. Amplification products were resolved by agarose gel electrophoresis. All reagents were sourced from Thermo Fisher Scientific (USA) and primers/probes were synthesized by Metabion International AG (Germany).

Positive PCR amplicons were purified using the QIAQuick purification Kit (Qiagen, Valencia, CA) according to the manufacturer`s instructions and Sanger sequencing of both strands was performed using ABI equipment (Applied Biosystems Inc., Foster City, CA). The resulting sequences were compared against reference databases using the NCBI BLAST tool to confirm species identity.

### Multilocus sequence typing (MLST) and data analysis

For MLST of *H. capsulatum* in the FFPE sample, we targeted three *H. capsulatum* MLST loci: alpha tubulin (Tub1) gene, ADP ribosylation factor (Arf) gene and H antigen precursor (anti-H) gene [15]. In this study, this method was slightly modified including a novel reverse primer for the anti-H gene, thus shorter anti-H gene fragment was targeted. In this study, the Tub 1 and Arf gene fragments were amplified using the primer pairs Tub1_F (5′-AGCTCCATTACAACAGCCAAT-3′), Tub1_R (5′-CTGCCGAGGAGGAACAGTTA-3′) and Arf_F (5′-CCATTGGTAAGTTCCTCGATTC-3′), Arf_R (5′-ACCGACGTCCCACACTGTAA-3′), respectively [15]. The PCR conditions were as follows: initial denaturation at 95 °C for 5 min, then 45 cycles of 95 °C for 15 s, 58 °C for 30 s; 72 °C for 30 s and a final extension step at 72 °C for 5 min. The anti-H gene fragment was amplified using the primers H-Anti_F (5′-CGTCGGGTACGCTCTCTC-3′) and H-Anti_Rw_lab_2 (5′-GTCATCTGGGTTGCTCACCA-3′) in the following conditions: initial denaturation at 95 °C for 3 min, then 40 cycles of 95 °C for 30 s, 56 °C for 30 s; 72 °C for 60 s and a final extension step at 72 °C for 5 min [15]. The final volume of the PCR sample for all reaction was 25 µL containing 10X DreamTaq buffer, 0.625 U of DreamTaq Hot Start polymerase, 200 µM of each dNTPs, 0.3 µM of each primer and 5 µL of DNA template.

After reactions, PCR products were subjected to electrophoresis in agarose gel to confirm the PCR results and sequenced on both strands as described earlier.

The Tub1, Arf and anti-H gene sequences from 182 *H. capsulatum* (*Ajellomyces capsulatus*) worldwide strains and samples were downloaded from the National Library of Medicine Nucleotide database (https://www.ncbi.nlm.nih.gov/nucleotide/; assessed on 08.11.2024). These sequences and the *H. capsulatum* sequences obtained from the cat tissue were trimmed to the sizes derived from the MLST primers used and concatenated. All the concatenated sequences were aligned by MUltiple Sequence Comparison by Log-Expectation (MUSCLE) and then analyzed by Neighbor-Joining (NJ) algorithm using Mega11 software [16].

## Results

### Cytology

**Fig. 2:**
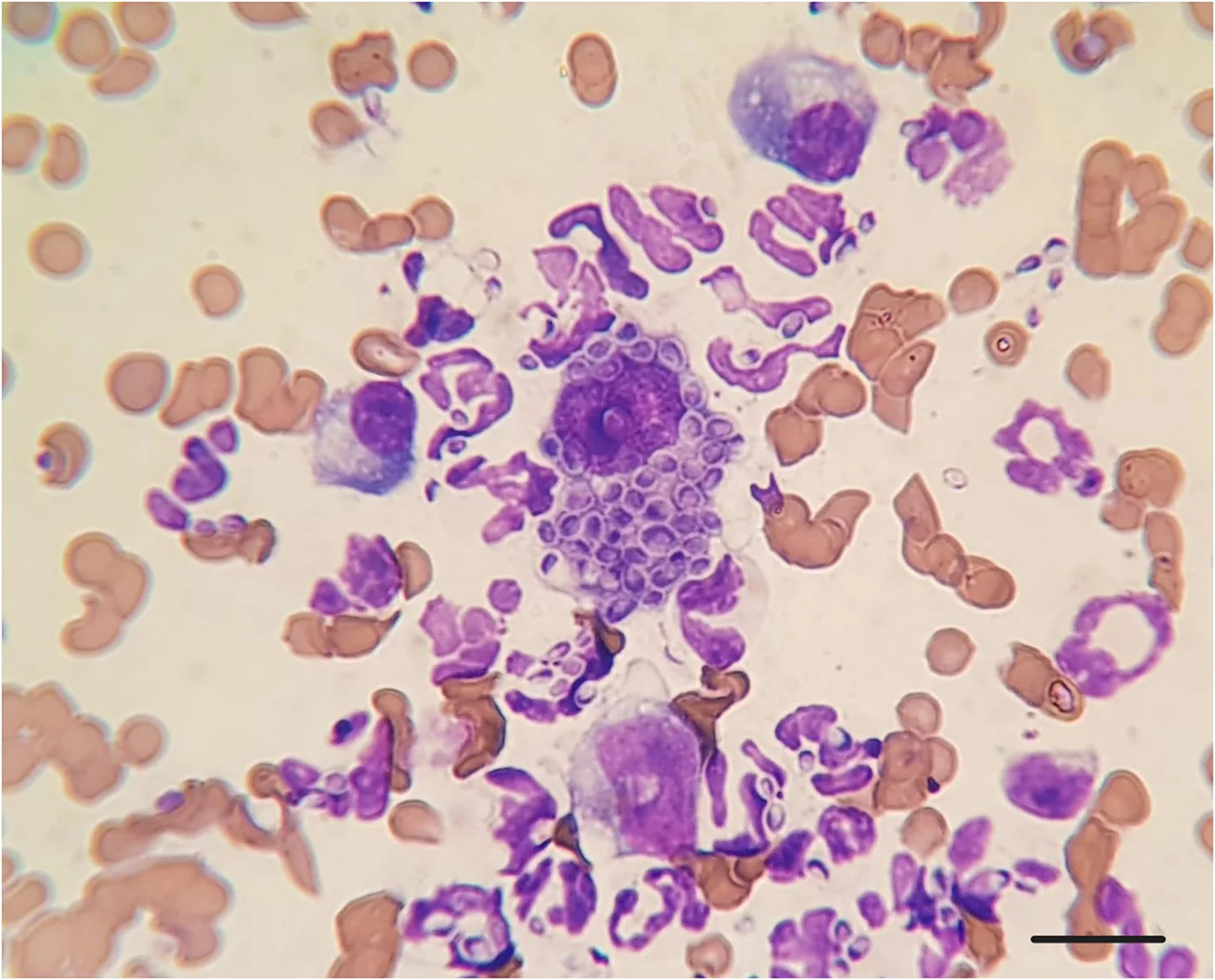
Fine needle aspirate from cutaneous lesion (Case 1). Cytological examination revealed numerous round to oval, 2–5 μm intracellular yeast organisms within macrophages and occasionally free in the background. Organisms show a thin, clear halo consistent with *Histoplasma capsulatum*. Partially broken neutrophils and red blood cells are also present. Romanowsky-type stain, original magnification ×1000, bar 10 μm

Fine-needle aspirates and impression smears were obtained from skin lesions in all three cats. In all three cases, cytology revealed moderate to severe granulomatous inflammation with numerous intra- and extracellular yeast-like organisms, approximately 3–5 μm in diameter, possessing a central basophilic nucleus and a thin perinuclear halo (Fig. 2). Morphological features on cytology smears were consistent with *H. capsulatum*.

### Histopathology and immunohistochemistry

**Fig. 3:**
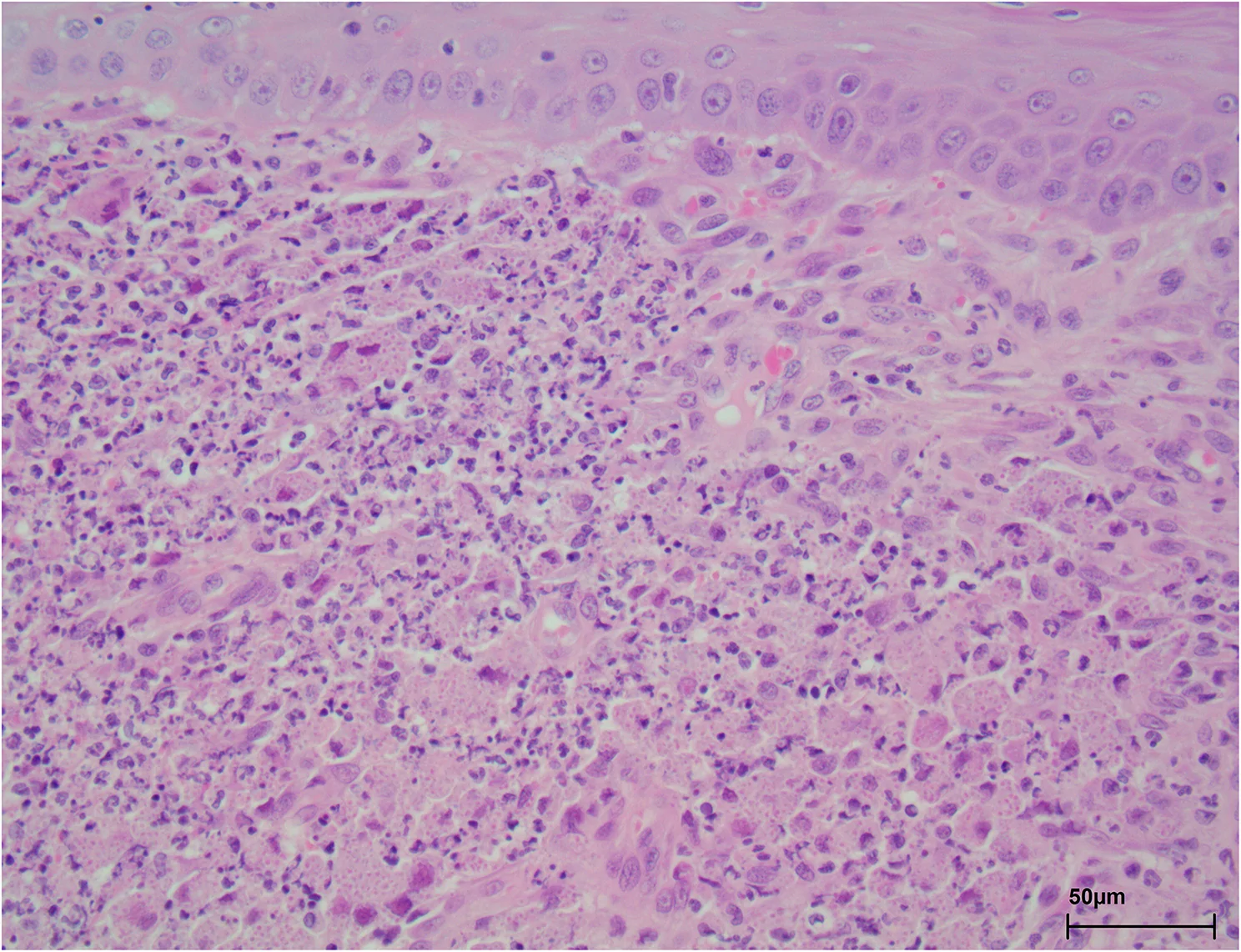
Histopathological section of a mucocutaneous junction lesion from cat, stained with haematoxylin and eosin (H&E). The section shows dense dermal inflammatory infiltrates composed of macrophages and neutrophils. There are myriads of small, 3–5 μm round to oval yeast-like organisms present in the cytoplasm of macrophages. Original magnification ×400

**Fig. 4:**
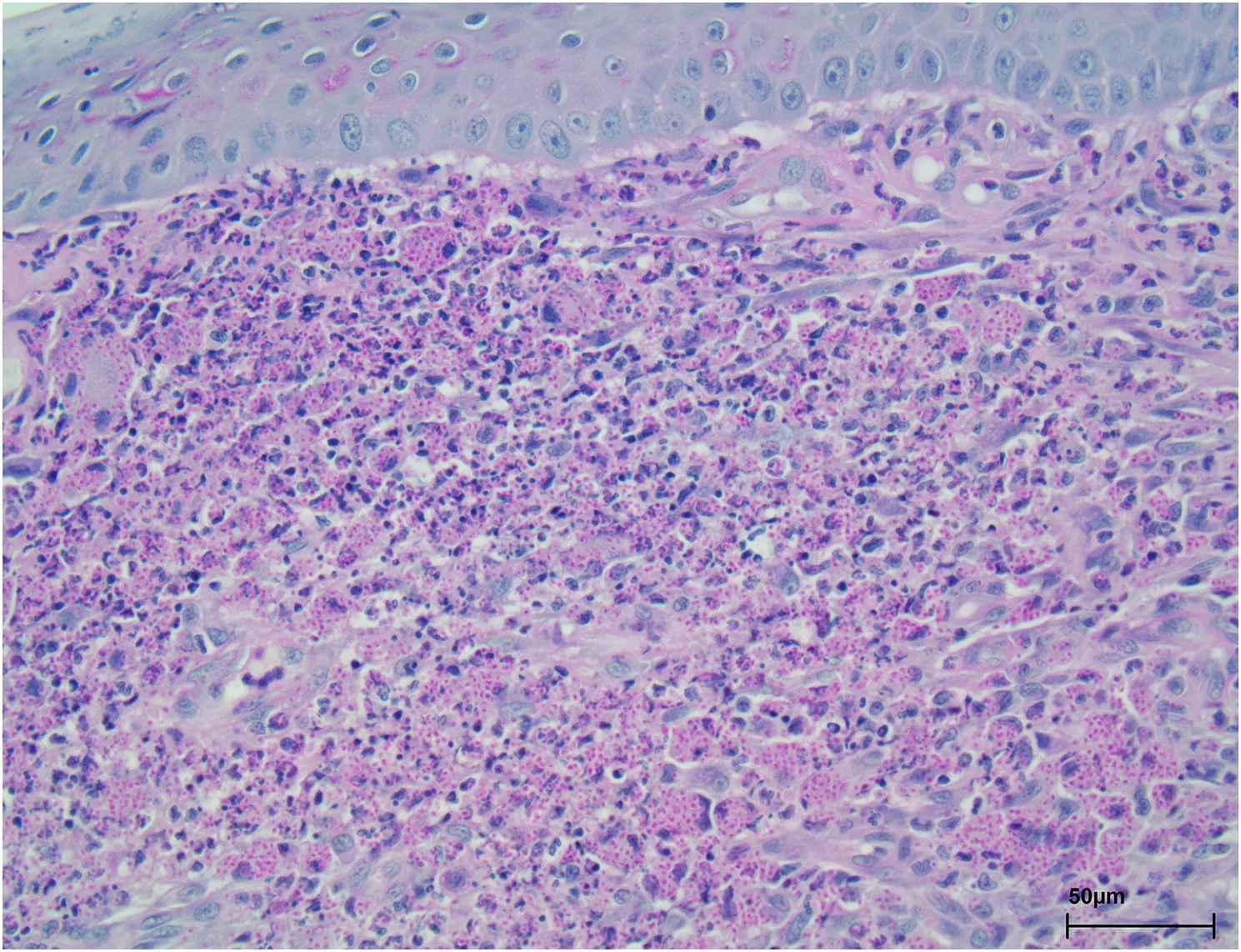
Histopathology of skin lesion from a cat (PAS stain). Numerous macrophages in the dermis contain 3–5 μm round to oval, magenta-staining yeast organisms with a clear halo, suggestive of *H. capsulatum*. PAS stain, original magnification ×400

Hematoxylin and eosin (H&E) staining of skin biopsies from Cases 1 and 3 demonstrated granulomatous to pyogranulomatous dermatitis with large numbers of intracellular and extracellular yeast organisms (Fig. 3). PAS stain confirmed presence of PAS positive, oval 3–5 μm in diameter fungal organisms surrounded by clear capsule (Fig. 4). Based on yeast morphology differentials were *Sporothrix* and *Histoplasma* species.

Immunohistochemistry (IHC) was performed on skin biopsies from Cases 1 and 3 at the Michigan State University Veterinary Diagnostic Laboratory. Fungal organisms were positive for *H. capsulatum*-specific immunostaining in both cases and negative for *Blastomyces* species. However, due to limited validation of the antibody against other fungal species, cross-reactivity with *Sporothrix* species could not be fully excluded.

### Serology

Serology testing was performed at MiraVista Diagnostics (Indianapolis IN, USA). In all three cases serum *H. capsulatum* antibody concentrations were 8.5, 8.2 and 8.5, respectively, placing them in the intermediate “gray zone” (reference: <8.0 negative; 8.0–9.9 intermediate; >10.0 positive). In Case 3, *H. capsulatum* antigen was detected in both serum (5.21 ng/mL) and urine (14.32 ng/mL), both falling within the diagnostic positive range.

### Molecular detection and sequencing

**Fig. 5:**
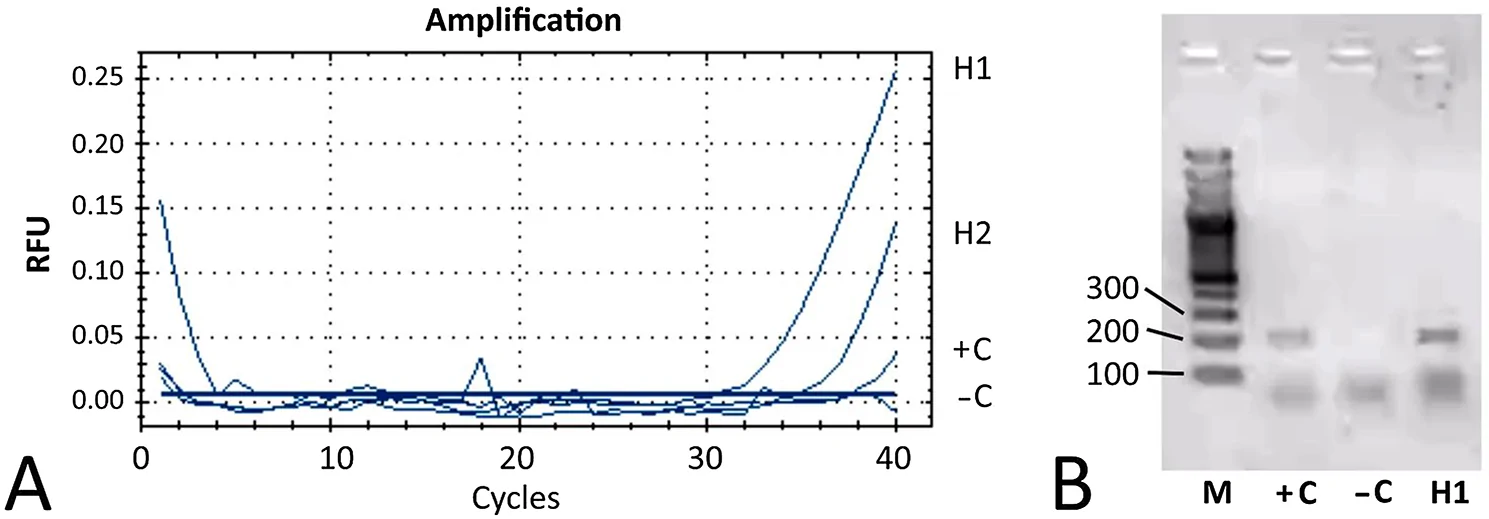
Molecular detection of *H. capsulatum* DNA. (A) qPCR amplification curves showing positive signals from H1 and H2 samples from cat 3, compared to positive (+ C) and negative (-C) controls. (B) Gel electrophoresis showing PCR products. Lane M = marker, +C = positive control, -C = negative control, H1 = DNA from cat 3 tissue

Paraffin-embedded biopsy material from Case 3 yielded amplifiable DNA. Paraffin blocks from Cases 1 and 2 were lost during transportation from USA to Latvia. Real-time PCR targeting the ITS1 region of the ribosomal DNA and nested PCR targeting the Hcp100 gene were both positive for *H. capsulatum* DNA (Fig. 5). Sequencing of the 210 bp Hcp100 gene fragment revealed 100% identity with multiple *H. capsulatum* reference isolates.

### Multilocus sequence typing (MLST) and phylogenetic analysis

**Fig. 6:**
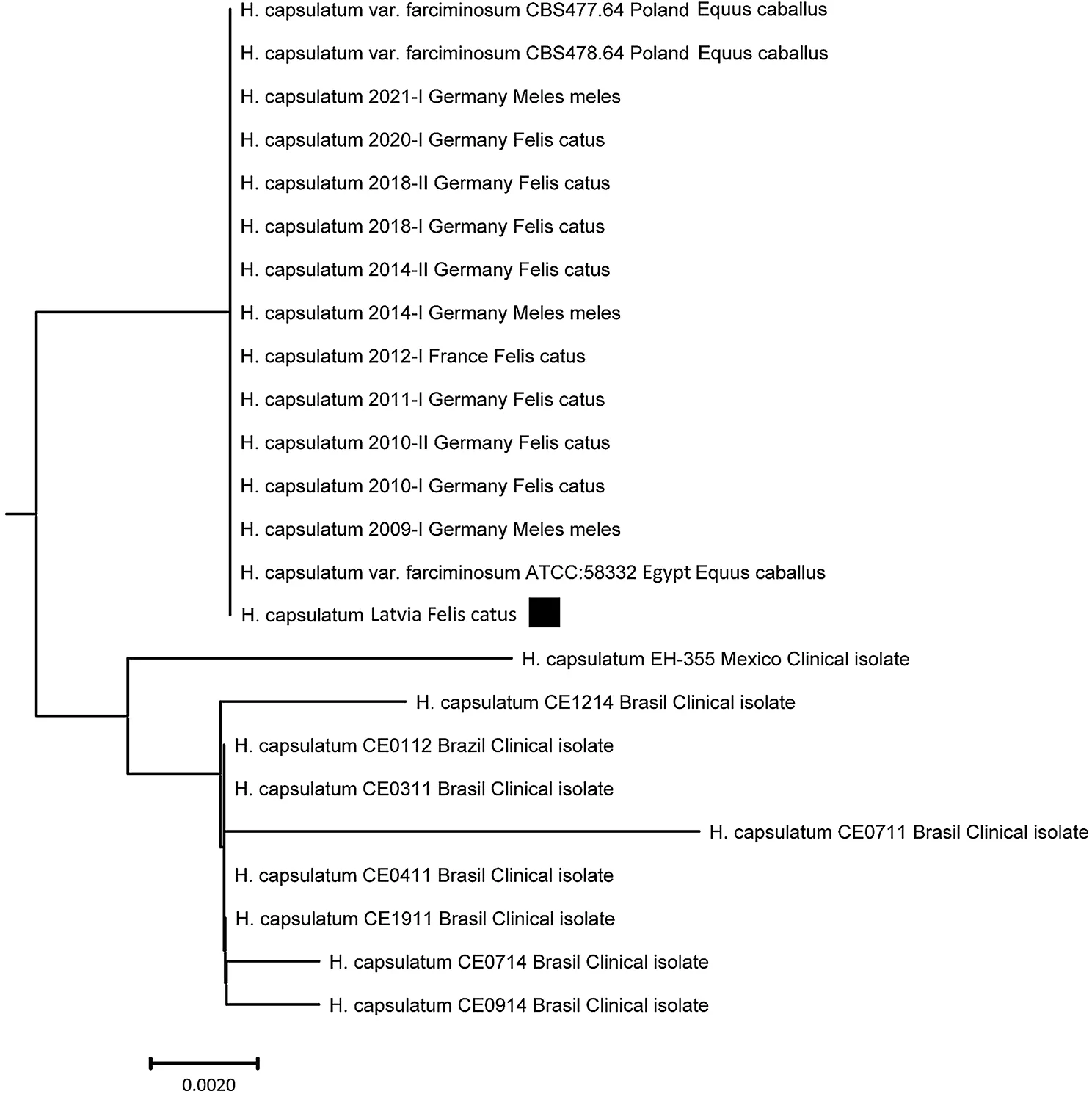
Phylogenetic placement of *H. capsulatum* isolate from case 3. MLST-based phylogenetic tree shows the Latvian isolate clustering with the Eurasian lineage of *H. capsulatum*, closely related to isolates from Germany and France

Three MLST loci (Tub1, Arf and anti-H genes) were successfully amplified and sequenced from Case 3 biopsy DNA. Sequence lengths after primer trimming were 165 bp (Tub1), 204 bp (Arf) and 202 bp (anti-H). The phylogenetic analysis was performed on the concatenated sequences (tub-arf-H-anti, 571 bp) and the Latvian cat isolate clustered with eleven feline and badger isolates from Germany and France and with three equine isolates from Poland and Egypt, including the reference strain ATCC58332 (*H. capsulatum var. farciminosum*) (bootstrap support 89%) (Fig. 6). Based on previously published classification, the equine ATCC58332 (H90) isolate belongs to the Eurasia geographic clade [17]. The obtained Hcp100, Tub1, Arf and anti-H gene sequences were deposited in GenBank under accession numbers PV256415 - PV256418.

## Discussion

This report describes three clustering cases of feline histoplasmosis from Latvia, including the first molecularly and phylogenetically confirmed case in the Baltic and Nordic region of Europe.

Clinically all cats presented with erosive or ulcerative skin lesions, while systemic signs were minimal or absent, such presentation is similar to the previously published cutaneous histoplasmosis case [6, 8]. This highlights the importance for general practitioners and veterinary dermatologists to consider fungal diseases such as histoplasmosis in the differential diagnosis of chronic, non-healing skin lesions—even in temperate regions not classically associated with endemic mycoses.

In our experience, routine cytological examination in such cases should be performed, as it has the potential to provide a timely rather than posthumous diagnosis and allows for initiation of the appropriate treatment [4, 7, 9]. Some authors suggest that histoplasma yeast can be difficult to observe on cytology without special stains; we did not experience any difficulties with routine cytology stains or the amount of yeast on the cytology slides obtained via fine needle aspiration or as impression smears but this might differ if other tissues are examined [9]. We also agree with numerous colleagues stating that the morphology of the histoplasma is typically uniform and suggestive of the diagnosis, the only offsetting fact being the subjective feeling “that histoplasmosis is not found in Europe” [7–9]. Diagnosing histoplasmosis in regions where it is not considered endemic poses several challenges, including a lack of awareness and scarcity of diagnostic tests; however, cytology and histopathology are frequently utilized diagnostic tests across Europe and are recommended as diagnostic tests for histoplasmosis [12]. In all three cases described here, cytology was the first diagnostic tool and provided a strong presumptive diagnosis. The characteristic intracellular yeast-like organisms were visible in routinely stained smears, highlighting the value of cytology as a rapid, accessible and cost-effective screening method [12]. Cytological features were consistent across all three cases, despite variations in lesion distribution, disease progression and previous treatment provided in primary care clinic.

Histopathology, while supportive of granulomatous inflammation, could not exclude sporotrichosis that can present with similar cutaneous lesions. Immunohistochemistry showed positive staining for *H. capsulatum* in cases 1 and 3, but due to limited validation and potential cross-reactivity with other dimorphic fungi (e.g. *Sporothrix species*), IHC diagnosis was not considered definitive. Publications list PAS and Gomori methenamine silver as special stains that could improve the sensitivity, but in case 3 differentiation between different fungal organisms was still relevant [2, 18].

Serological testing yielded equivocal antibody levels in all three cases, likely reflecting early infection or variable immune response. The usefulness of antibody testing has been questioned in several publications, and we agree that antigen testing in serum and urine proved more informative, but the possible influence of a more pronounced disease or higher antigen concentration in case 3 cannot be excluded [1, 6, 19, 20].

The successful use of PCR and sequencing on formalin-fixed paraffin-embedded tissue provided a definitive diagnosis in the third case reported here. The addition of MLST allowed precise phylogenetic placement of the strain and demonstrated its genetic similarity to other Eurasian isolates, supporting autochthonous infection. This case exemplifies the value of molecular diagnostics in fungal disease confirmation, especially in non-endemic settings where clinical suspicion may be low and available diagnostic tests are limited. Diagnostic (serological, PCR) test availability is limited in European laboratories, resulting in increased expenses and time needed for definitive diagnosis. If diagnostic material is shipped to USA, it can cause delays and potential problems, including the loss of material in transit as in our case when FFPE tissues used for IHC were lost for further PCR and phylogenetic analysis. Strains isolated from Europe often lack phylogenetic analysis, we have addressed this to the best of our ability [6]. The present study includes cytological, histopathological, serological and molecular findings and case 3 was confirmed via MLST that is suggested to be the best method to increase understanding of the molecular epidemiology of histoplasmosis across Europe [6, 21]. Phylogenetic analysis placed the isolate within the Eurasia clade of *H. capsulatum*, grouping it with strains previously identified in Germany, France and Poland. This finding supports a local source of infection and reinforces the geographic expansion of histoplasmosis in Europe.

Three cats that we describe were male (two intact, one castrated), literature does not suggest any sex predilection, while outdoor access is a definitive risk factor and was present in all of our cases [8, 11, 18]. None of our cats had a history of travel which is described as a risk factor, and that could explain subclinical cases in a non-endemic area [9, 10]. All three cats described here were ginger, one was long haired, two were short haired, if coat colour and length could play a role in the susceptibility for this infection is unknown. One of the cats was FIV-positive, that has a negative effect on immunity, potentially increasing chances of infection, but retroviral infections have not been associated with increased seropositivity against histoplasma [22].

From an epidemiological perspective, our findings indicate the likely environmental presence of *H. capsulatum* in Latvia. Up-to-date reported European feline histoplasmosis cases have been sporadic, our cases show signs of clustering, both geographically (same town) and temporally (within 2 consecutive years, during late spring and early autumn). Although the environmental reservoir was not directly investigated, the clustering of cases and local acquisition suggest that further surveillance is warranted as fungi might be introduced also with fertilizers or through migrating animals [23]. Climate change is suspected to play a role as in other infections that are newly being diagnosed in new, more northern areas [9].

We do not know of any autochtonous human histoplasmosis cases in Latvia, rare cases that have been diagnosed are related to travel history to endemic areas. Although there is potential for zoonotic risk, no direct transfer from infected animals to humans has been described [9].

This study has several limitations. We have not located the exact source of infection for our indoor - outdoor living cats. Literature search suggests that digging in outside soil, access to unfinished basements or construction sites, living with pet birds, exposure to pigeons, chicken, bats as well as owners diagnosed with histoplasmosis and access to indoor potted plants could all be potential sources of infection [10, 12]. Two of our cats were living in the close neighbourhood to the same broken shed where bats had been observed, but a detailed examination of the shed was not performed and bat droppings were not collected for evaluation. We extracted DNA from paraffin embedded tissues, this likely had a negative influence on the length of the DNA sequence we were able to obtain, frozen tissue usually yields longer DNA strands that would be better suited for phylogenetic analysis [21]. Only one case was confirmed by molecular and phylogenetic methods. Biopsy sample from one cat was lost during shipment and one of the cats was lost for follow-up, thus the efficacy of the treatment could not be assessed. Additionally, the IHC method that was used for detection of *Histoplasma* species antigen was not rigorously validated and cross-reactivity with other morphologically similar fungal organisms could not be completely excluded.

Nevertheless, the consistency of cytological findings across all cases and the robust molecular confirmation in Case 3, provide strong evidence for histoplasmosis as an emerging or underrecognized disease in northern Europe.

## Conclusions

This case series provides compelling evidence that *H. capsulatum* may exist and be underdiagnosed in northern Europe. The consistent cytological findings across all three cases, combined with molecular confirmation and phylogenetic placement within the Eurasia clade, strongly support autochthonous infection in Latvia. These findings emphasize the value of cytology as a practical and accessible diagnostic tool and encourage veterinary clinicians to include histoplasmosis in differential diagnoses of chronic dermatologic lesions in cats—even outside classical endemic regions.

## Data Availability

No datasets were generated or analysed during the current study.

## Declarations

### Prior publication

Data have not been published previously.
